# An injectable acoustic transmitter for juvenile salmon

**DOI:** 10.1038/srep08111

**Published:** 2015-01-29

**Authors:** Z. D. Deng, T. J. Carlson, H. Li, J. Xiao, M. J. Myjak, J. Lu, J. J. Martinez, C. M. Woodley, M. A. Weiland, M. B. Eppard

**Affiliations:** 1 Pacific Northwest National Laboratory, P.O. Box 999, Richland, WA 99352

## Abstract

Salmon recovery and the potential detrimental effects of dams on fish have been attracting national attention due to the environmental and economic implications. In recent years acoustic telemetry has been the primary method for studying salmon passage. However, the size of the existing transmitters limits the minimum size of fish that can be studied, introducing a bias to the study results. We developed the first acoustic fish transmitter that can be implanted by injection instead of surgery. The new injectable transmitter lasts four times longer and weighs 30% less than other transmitters. Because the new transmitter costs significantly less to use and may substantially reduce adverse effects of implantation and tag burden, it will allow for study of migration behavior and survival of species and sizes of fish that have never been studied before. The new technology will lead to critical information needed for salmon recovery and the development of fish-friendly hydroelectric systems.

Five species of Columbia River Basin salmonids (*Oncorhynchus spp*.) and 13 of their 19 evolutionary significant units have been listed for protection as “threatened” or “endangered” under the Endangered Species Act (ESA) due to the significant population decline^1^,^2^,^3^. Salmon recovery and the potential detrimental effects of hydroelectric dams and habitat loss on fish survival have been attracting significant national attention in the last two decades due to the environmental and economic implications^4^,^5^,^6^,^7^,^8^. To aid in the recovery, it is critical to develop better technologies for increased understanding of the behavior of migrating salmonids through impounded river systems in order to optimize the designs and operations of dam passage facilities and improve the survival rates of salmonids^9^,^10^.

In 1987, the U.S. Army Corps of Engineers (USACE) initiated a program to assess the engineering and biological performance of the Federal Columbia River Power System (FCRPS). Emphasis was placed on making structural and operational changes to FCRPS dams that would enhance the survivorship of juvenile salmon migrating downriver to the ocean^11^. The process of increasing survivorship during dam passage required studies to evaluate the biological and engineering performance of the existing structures and operation, development of alternatives that could improve dam passage conditions, and implementation of the highest priority alternatives. This was followed by an assessment of the changes in dam passage survival, produced by modifications in dam structures and operations^12^.

Over the years a number of different survival assessment methods were tested, with selection of telemetry as the best available technology to provide the dam passage metrics needed to evaluate the effectiveness of dam passage improvement alternatives. Initially, commercially available implantable radio and acoustic telemetry transmitters and dam-mounted receivers were used^13^,^14^. The challenge was that the available transmitters were large and, in the case of radio telemetry, required that an antenna be passed through the body of the fish so that it trailed bare in the water outside of the body of the fish, while the transmitter remained within the tagged fish's stomach or abdomen (depending upon the method of implantation used to tag study fish).

Eventually, acoustic telemetry became the preferred telemetry method used to obtain measures of fish dam passage survival and other migration behavior metrics. However, the available transmitters were large, limiting tagging of study fish (implantation of a transmitter) to the largest portion of the out migrating population^15^. In addition, the implantation surgeries required extensive handling of study fish, which was thought to potentially negatively bias fish survival measures and other migratory behavior metrics. The USACE initiated a project with Pacific Northwest National Laboratory (PNNL) and The National Marine Fisheries Service to reduce the size and weight of acoustic transmitters, transferring any technology developed to the private sector through competitive procurement actions targeted to provide commercial volumes of acoustic micro-transmitters that were smaller in size and weight than those that were previously commercially available. The result was the Juvenile Salmon Acoustic Telemetry System (JSATS), consisting of acoustic micro-transmitters and autonomous and cabled receivers^16^,^17^,^18^.

The initial JSATS micro-transmitters were smaller in size and weight than their commercially available predecessors but still required surgeries for implantation in the abdomens of study fish. Study results indicated that the new transmitters and advancements in surgical procedures would provide improvements in dam passage survival studies^15^. Additional investigation of micro-transmitter designs, particularly evolving micro-battery technology^19^, showed that significant reductions in the cost of survival studies and in fish handling effects could be obtained by additional reductions in micro-transmitter size and weight, and by designing a transmitter that could be implanted in a fish's abdomen by injection using a syringe and needle as opposed to a surgery requiring an incision, hand insertion of a transmitter, and sutures. After careful consideration of potential benefits and a technology feasibility assessment, development was initiated to design and test a JSATS micro-transmitter with the same operating characteristics of larger transmitters, while being considerably smaller and implantable by injection.

## Results

**Figure 1:**
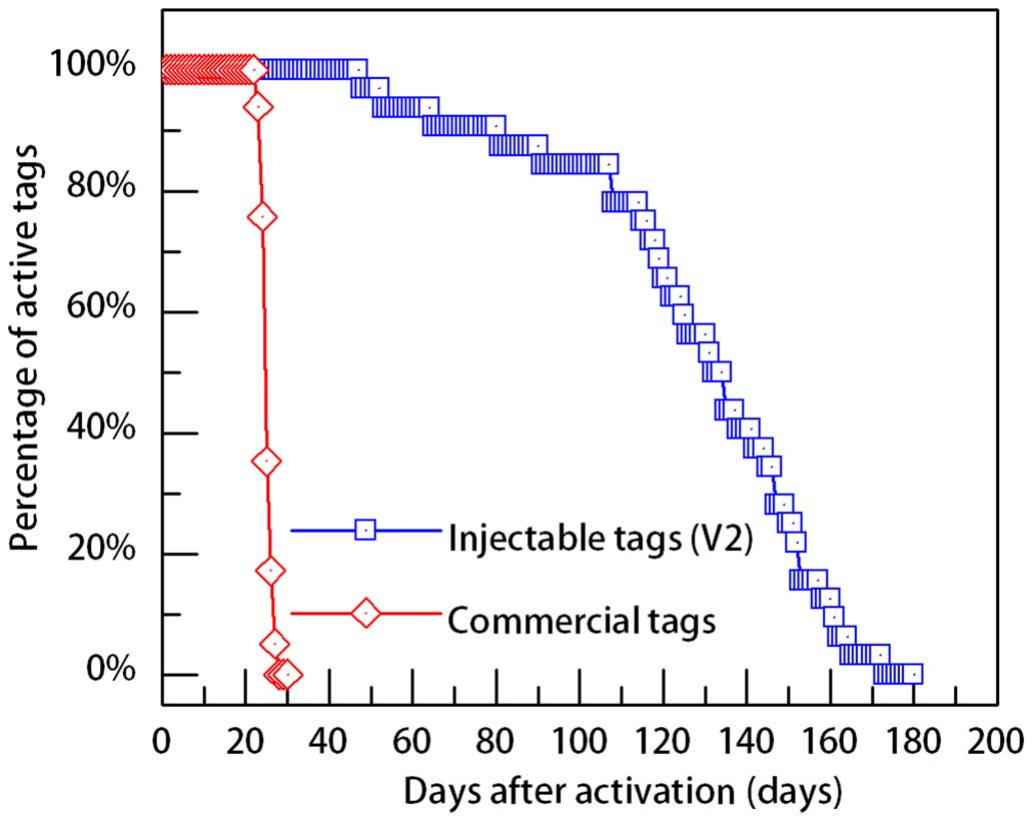
Tag life comparison between the injectable JSATS transmitter (V2) and the smallest commercial JSATS transmitter at 3-second ping rate.

The injectable JSATS transmitter consists of three main components: a PZT (lead zirconate titanate) tube transducer which emits acoustic signals, an electronic circuit board which contains the controlling circuitry, and a lithium/carbon fluoride battery which powers the entire transmitter. Two design variants of the injectable transmitter that use two different types of drive circuits were developed, named “V1” and “V2”, respectively. The two transmitter designs have identical shape, weight, and dimensions. The main functional difference between the two is that the V1 design offers a configurable source level up to 158 dB (re: 1 μPa at 1 meter), while the V2 design has a fixed source level of 155 dB (ref: 1 μPa at 1 meter) and an exceptionally long tag life (e.g. >100 days at a 3-sec ping rate, compared to ~20 days for the V1 design and the commercially available acoustic fish transmitter at the same ping rate and similar source level). A tag life comparison between the V2 transmitter and the smallest commercially available acoustic fish transmitter is shown in Figure 1. These tag life results for both transmitters were based on a sample size greater than 30 transmitters.

**Figure 2:**
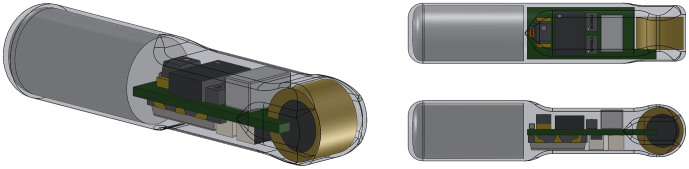
A schematic of the injectable JSATS acoustic transmitter showing the transmitter from three different angles.

**Figure 3:**
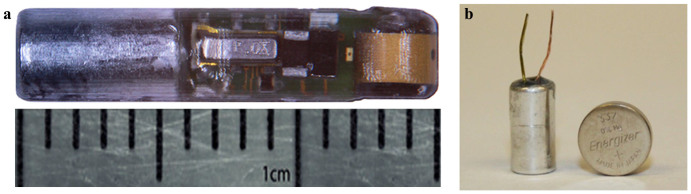
Photographs of the injectable transmitter and the PNNL-developed micro-battery used by the injectable transmitter: (a) the injectable transmitter; (b) the micro-battery standing next to a commercial 337 button-cell battery which is used by the existing commercial JSATS transmitters. (photos by H.L.)

Figure 2 is a schematic of the transmitter, showing from three different angles the three main components encapsulated by epoxy in a primarily cylindrical body. For weight reduction as well as acoustic beam pattern optimization purposes, the front portion containing the PZT and circuit board has a smaller cross section than the rear portion. The transmitter is 15.0 mm long and has a diameter of 3.38 mm, which allows it to be injected using an 8-gauge needle. The dry weight of the transmitter is 217 milligrams, about 30% lighter than the smallest commercially available acoustic fish transmitter^16^, offering significant tag burden reduction to the tagged fish. Tag burden is defined as the ratio of the dry weight of the transmitter relative to the dry weight of the fish in which it is implanted. A photograph of the prototype injectable transmitter is shown in Figure 3a.

Both transmitter designs use a microcontroller as the primary controlling element and an infrared sensor as the receiving interface for programming and activation. Thus, it can be conveniently programmed or activated using an infrared “blaster” connected to a personal computer in just a few seconds. Once activated, the transmitter broadcasts a unique acoustic identification code (“tag code” hereinafter) at a pre-set pulse rate interval (PRI). In addition, the transmitter has the following functions:

- The source level (V1 only) and PRI are configurable by the user
- Automatic charging length adjustment to maintain constant acoustic signal strength as the battery discharges
- The ability to alternately transmit two different 16-digit binary tag codes, allowing for more than 4.2 billion unique identifiers
- The ability to embed temperature measurements into the tag code
- The ability to hibernate for a user specified period of time before starting transmission
- An ultra low power mode for maximum shelf life

To meet the power, size, and weight requirements of the new injectable JSATS transmitter, a novel lithium/carbon fluoride (Li/CF_x_) micro-battery (MB306) was developed. Compared to the traditional silver oxide button-cell batteries (SR416), which are commonly used in existing small acoustic fish tags, Li/CF_x_ batteries have the advantages of high power density, high average operating voltage (3.1–3.4 volts), long shelf life, and a wide operating temperature range^20^. Theoretically, carbon monofluoride (CF_x_ with x = 1) has a practical high energy density of up to 650 Wh/kg in an envelope cell^21^, more than four times higher than that of SR416, which has an energy density less than 150 Wh/kg.

The MB306 cell (Figure 3b) utilizes a jelly-roll structure to enable a cylindrical package shape so that it may be compatible with the cylindrical capsule body of the injectable transmitter. A unique lamination process is used to allow for high loading of the active material, maximizing the electrode area in the very limited space dictated by the small transmitter package. This design offers the flexibility to readily increase battery capacity by extending the length of the cell when longer service life or higher acoustic signal strength is desired, which may also be the case for transmitters targeting larger fishes. The final battery package is 6.0 mm long, 3.0 mm in diameter and weighs approximately 70 milligrams^20^. In addition, the flexible cell design can be easily adapted into a flexible transmitter body for other fish species, such as juvenile lamprey.

**Figure 4:**
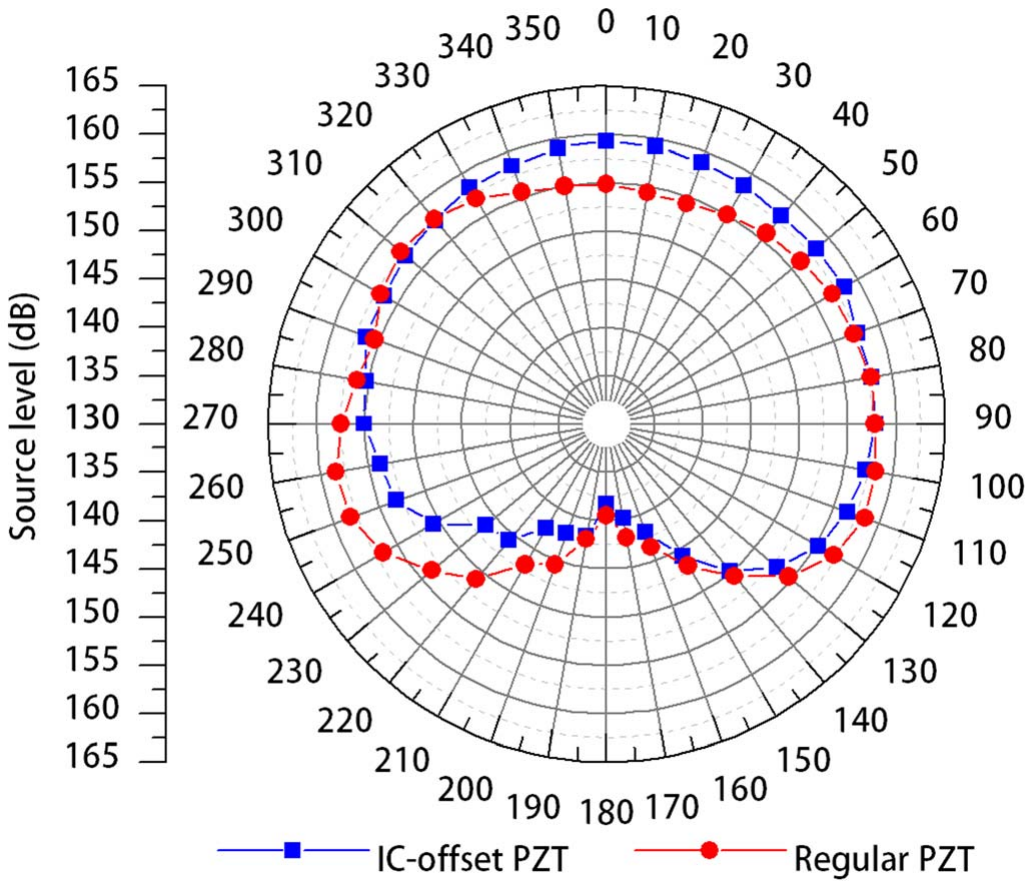
Beam pattern comparison between the injectable JSATS acoustic transmitter which uses an IC-offset PZT tube transducer and an existing JSATS transmitter which uses a regular PZT tube transducer.

The piezoelectric transducer used in the injectable transmitter is a PZT-5H ceramic tube which operates in hoop mode (or “breathing mode”). The PZT tube is polarized through its wall thickness direction. During operation, it is excited with an alternating current signal and thus vibrates radially in a breathing motion. As a result, acoustic signals are primarily radiated 360° from the wall surface of the PZT. The dimensions of the tube were selected such that the tube would have a hoop mode resonance frequency of 416.7 kHz, the application frequency of the JSATS, and would fit the 3.4 mm inner diameter of an 8-gauge hypodermic needle. The tube geometry was used to achieve an omnidirectional acoustic beam pattern. In reality, however, since the rest of the transmitter body is positioned behind the PZT, thus blocking the acoustic signals emitted from the back of the PZT, the overall acoustic beam pattern is only uniform within the front 180° and the acoustic signal strength towards the rear of the transmitter is much weaker, which is the case for all existing commercial acoustic fish transmitters. The acoustic energy emitted toward the rear of the transmitter can be considered largely “wasted” as it will have a much lower probability of being detected by the acoustic receivers placed on the hydroelectric dams. In order to more efficiently use the total energy delivered to the PZT, the PZT tube transducer for the injectable transmitter uses a special off-center design. Namely, the center of the inner circumference (IC) of the tube is purposely offset from that of the outer circumference such that the thinnest portion of the PZT tube is positioned at the front end of the transmitter and the thickest portion is positioned facing the circuit board (Figure 2). Compared to a regular PZT tube, when supplied with the same amplitude of voltage through the tube's wall thickness direction, the IC-offset design allows the front half of the PZT tube to be driven harder than the rear half due to the smaller wall thickness, thus directing more input energy towards the front of the transmitter. The injectable transmitter has enhanced source level in the front 180° range (the top half of the beam pattern) and reduced source level in the rear 180° (Figure 4).

**Figure 5:**
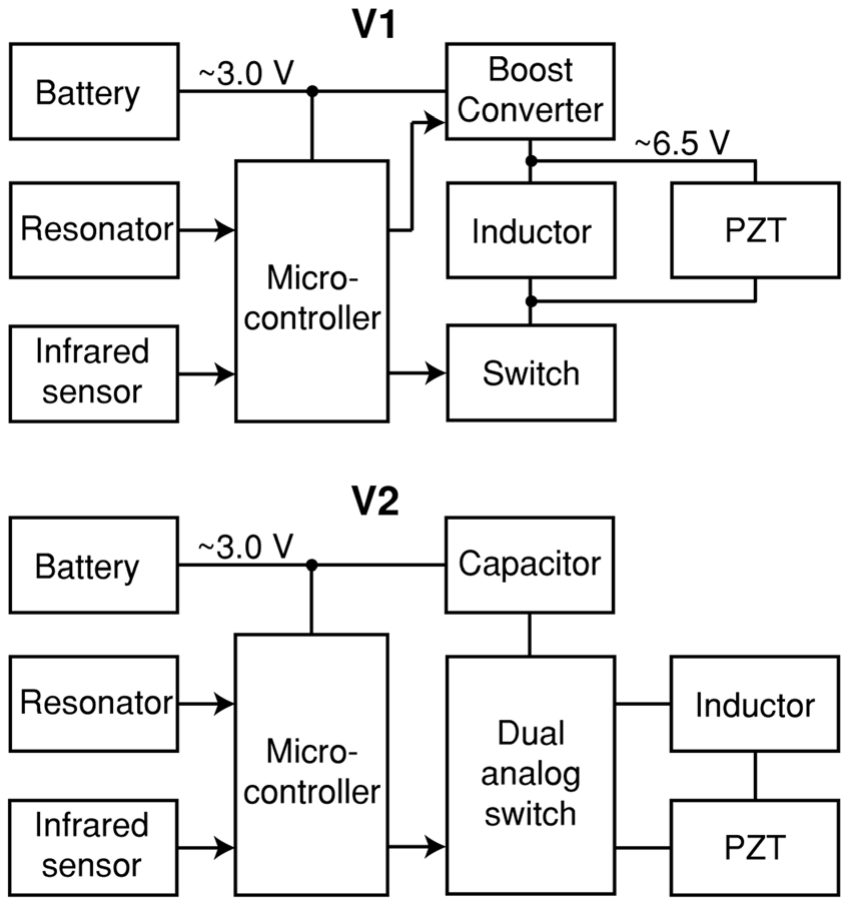
Block diagrams of how the two design variants of the injectable JSATS acoustic transmitter operate.

Figure 5 provides the block diagrams for the V1 and V2 designs. Both designs use a small microcontroller to generate the coded waveforms needed to drive the PZT transducer. A ceramic resonator at the input of the microcontroller generates a precise clock signal for controlling the modulation frequency. An infrared sensor allows the user to load the tag code, PRI, and other parameters into the microcontroller. In the V1 design, one output of the microcontroller controls a boost converter circuit, which transforms the battery voltage into a higher driving voltage for the PZT. Another output modulates the opposite side of the PZT at the specified frequency. To further increase the effective driving voltage, an inductor inside the drive circuit establishes a resonance with the PZT. In the V2 design, the boost converter is omitted. The microcontroller instead controls two analog switches that apply the battery voltage onto either side of the PZT. A higher quality but physically larger inductor is used to mitigate the lower driving voltage that results. Finally, a bulk capacitor smooths the large current pulse that occurs during transmission to prevent damage to the battery.

In the summer of 2013, approximately 1000 of these injectable transmitters (V1) were fabricated at PNNL. A total of 700 of these transmitters were implanted by injection using 8-gauge needles in run-of-the-river subyearling Chinook salmon in a field evaluation study in the Snake River of Washington State. Important passage data of juvenile salmonoids implanted with the injectable transmitters was successfully collected and used to evaluate their survival and migration behavior through the FCRPS.

## Discussion

The development of the JSATS system was driven by the need for a telemetry system that could be used to observe migratory behavior and estimate survival of juvenile salmonids passing through USACE dams during their downstream migration through the Columbia and Snake rivers. The injectable JSATS compatible acoustic transmitter meets or exceeds the operational specifications of previous JSATS acoustic transmitters^20^. The operational performance of the new transmitters has been documented under laboratory and field conditions. In addition, the new transmitter achieves the desired biological benefits allowing use in smaller fish while reducing the handling time of study fish during transmitter implantation using the injection method instead of surgical implantation. Reduction in handling and physiological trauma during implantation will result in study fish that behave and survive more like untagged downstream migrants, providing better assessments of measures taken to improve dam passage and downstream migration conditions. There is evidence showing that juvenile fish surgically implanted with transmitters behave somewhat differently and have lower survival than fish that are not bearing transmitters^22^. The possibility of differences in behavior and survival of tagged and untagged downstream migrants has been accepted because estimates of survival and observations of behavior of tagged fish would provide conservative estimates relative to the response of untagged migrants. In this way, the use of potentially negatively biased measures of survival and behavior would lead to increased benefits for untagged migrants as changes to dam structures and operations meet federally mandated performance criteria^12^. Reductions in handling effects on behavior and survival should provide estimates that have less bias and are more accurate of the response of the general untagged migrant population to measures taken to decrease the effects of dams on their survival during migration to the ocean.

Data exists in one area to assess the benefits of a smaller transmitter. Tag burden affects the behavior of the tagged fish and its response to exposures during dam passage, such as rapid decompression during passage through turbines and spill^20^. Guidelines for implantation of transmitters in juvenile salmon limit the ratio of transmitter to fish dry weights to a maximum 0.05 and encourage much lower ratios^23^. High ratios are thought to result in tagged fish being more shallowly distributed than untagged fish, and tagged fish being more susceptible to mortal injury during rapid decompression than untagged fish^22^. It is also suspected, but unproven, that surgical implantation of transmitters reduces the fitness of test fish relative to untagged fish, resulting in conservative estimates of dam passage survival. Laboratory and field studies are underway to assess the performance of juvenile salmon implanted by injection with new micro acoustic transmitters against those surgically implanted with larger acoustic transmitters.

The combination of very small acoustic transmitters and implantation of the transmitters by injection opens up the possibility of using acoustic telemetry to study fish species or age groups that were too small or too fragile to tag using larger transmitters or surgical implantation method. Although only tested at a small scale under laboratory conditions to date, implantation of micro-transmitters by injection can be accomplished underwater with minimal handling of test fish. If techniques can be developed to successfully capture and implant transmitters in fish underwater with minimal handling, the use of acoustic telemetry to study various species of freshwater and marine physoclistous fish, which are difficult to bring to the surface for implantation, will be possible.

## Methods

### Fabrication of the injectable JSATS acoustic transmitter

**Figure 6:**
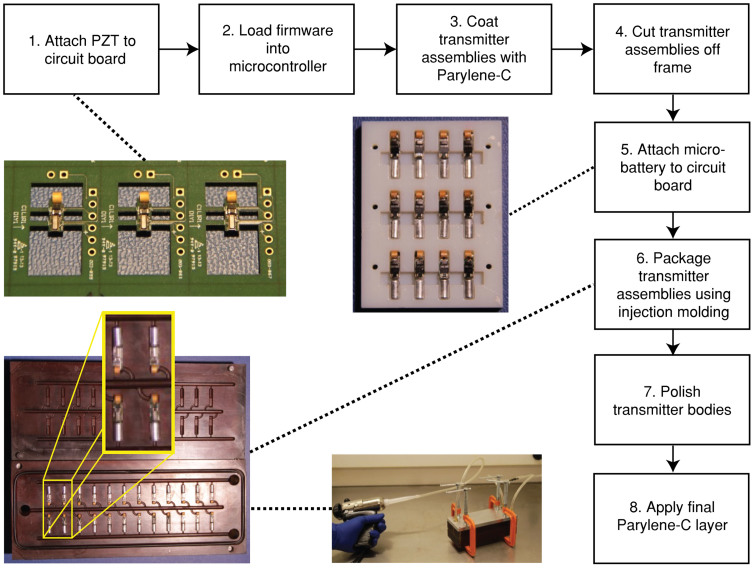
The fabrication process of the injectable JSATS acoustic transmitter. (photos by H.L.)

The injectable transmitter is fabricated through the steps illustrated in Figure 6. The IC-offset PZT transducer is first attached to the circuit board using a conductive silver epoxy, followed by loading the transmitter firmware into the on-board microcontroller. To prevent the electronic components on the circuit board from potential damages during the later fabrication steps, a 25-μm thick Parylene-C protective layer is coated onto the entire assembly. The MB306 micro-battery is then attached to the circuit board using the conductive silver epoxy. To encapsulate the transmitter with an insulating epoxy, the assembly is placed into a specifically designed plastic (Ultem 1000, SABIC, Pittsfield, MA, USA) injection mold (shown in Figure 6). An insulating epoxy (3M Scotchcast Electrical Resin 5, 3M, St. Paul, MN, USA) is injected into the mold through an epoxy inlet on the mold by an air sealant gun and discharges through the outlets on the other end of the mold. The epoxy is kept continuously flowing until no air bubbles are observed exiting the mold. After injection, the mold is left standing at room temperature overnight for the epoxy to cure. The transmitters are then removed from the mold and polished to obtain a smooth finish so that no tissue irritation by the transmitter will be caused due to rough edges after it is implanted into fish's body cavity. Lastly, another 25-μm layer of Parylene-C is coated on the exterior of the transmitter, which serves as a water-proof as well as biocompatible layer.

### Source level measurement

The source level measurements were conducted in an acoustic water tank filled with fresh water. The tank was 1.26 m long, 0.95 m wide, and 0.90 m deep. The interior walls, the tank bottom, and the bottom of the tank cover were lined with a 26-mm-thick layer of a sound absorbing material (Aptflex F48, Precision Acoustics Ltd., Dorchester, Dorset, UK), which provides excellent reduction of ultrasound reflection in the sub-MHz frequency range, minimizing the impact of echoes and noises inside the tank on the source level measurements. The transmitter and a receiving hydrophone (Model SC 001-2008-0404, Sonic Concepts, Bothell, WA, USA) with a 10.6-dB gain were positioned 0.5 m underwater and one meter apart. Prior to the measurements, the receiving hydrophone was calibrated with an omnidirectional broadband projector hydrophone (Model TC-4034, Reson A/S, Slangerup, Denmark)^24^. The transmitter was mounted on a motion control unit so its movement could be three-dimensionally controlled by a computer. The transmitter was oriented the same way as it would be in the fish's body cavity, which is that the tube axis of its PZT transducer was perpendicular to the water surface and the length direction of the transmitter is in line with the receiving hydrophone as the starting position. During the measurements, the transmitter disseminated an actual JSATS tag code at a PRI of 0.5 seconds. The motion control unit rotated the transmitter about the vertical axis at a 10-degree interval. The average of the source level values at each angle of the front 180° (0° is defined as the transmitter orientation in which the PZT was closest to the hydrophone) was used as the representative source level of the transmitter.

## Author Contributions

Z.D., T.C. and M.E. designed and directed the study; H.L., J.X., M.M., J.L., J.M. and Z.D. conducted the engineering experiments; C.W. and M.W. developed the implantation method; Z.D., H.L. and T.C. wrote the manuscript. All authors reviewed the manuscript.
